# Identifying patterns of high intraoperative blood pressure variability in noncardiac surgery using explainable machine learning: a retrospective cohort study

**DOI:** 10.1080/07853890.2025.2537920

**Published:** 2025-07-24

**Authors:** Zheng Zhang, Jian Wu, Yi Duan, Linwei Liu, Yaru Liu, Jinghan Wang, Li Xiao, Zhifeng Gao

**Affiliations:** a Department of Anesthesiology, Beijing Tsinghua Changgung Hospital, School of Clinical Medicine, Tsinghua Medicine, Tsinghua University, Beijing, China; b School of Artificial Intelligence, Beijing University of Posts and Telecommunications, Beijing, China; c Institute of Computing Technology, Chinese Academy of Sciences, Beijing, China

**Keywords:** Machine learning, blood pressure variability, Anesthesia management

## Abstract

**Background:**

High intraoperative blood pressure variability (HIBPV) is significantly associated with postoperative adverse complications. However, practical tools to characterize perioperative factors associated with HIBPV remain limited. This study aimed to develop explainable supervised machine learning (ML) models to classify patients with HIBPV and to identify structural perioperative patterns associated with HIBPV through model interpretation.

**Materials and Methods:**

This retrospective cohort study analyzed 47,520 noncardiac surgery cases from Beijing Tsinghua Changgung Hospital. We applied four ML algorithms—Extreme Gradient Boosting (XGBoost), Random Forest (RF), Light Gradient Boosting Machine (LightGBM), and Logistic Regression (LR)—to classify patients with or without HIBPV. The overall population and each age subgroup (pediatric, adult, elderly) underwent independent 70/30 train-test splits for model development. Model performance was assessed using the area under the receiver operating characteristic curve (AUROC). SHapley Additive exPlanations (SHAP) values were used to interpret model outputs and assess feature importance.

**Results:**

Among 47,520 noncardiac surgeries, 1,996 (4.2%) were classified as HIBPV. XGBoost and RF achieved the best performance, with AUROC values of 0.85 (95% confidence intervals (CI): 0.84–0.86) and 0.84 (95% CI: 0.82–0.85). Intraoperative average heart rate (HR) and bispectral index (BIS) were the most influential variables. In patients aged 50 ∼ 70, higher sevoflurane dosage was associated with reduced HIBPV risk. Among hypertensive patients, elevated intraoperative blood calcium (>1.10 mmol/L) was associated with increased HIBPV risk.

**Conclusion:**

The models enabled accurate classification of HIBPV cases and highlighted key discriminative perioperative variables through SHAP-based interpretation. Intraoperative HR and BIS were significant contributing factors. Moreover, interactions between sevoflurane and age and between hypertension and calcium levels may inform individualized hemodynamic management strategies.

## 1. Introduction

Blood pressure variability (BPV) refers to the fluctuations in blood pressure over a defined period [1,2]. Notably, it serves as a valuable complement to single-point blood pressure measurements [1,2]. Since the concept was first introduced by Hammarstrom in 1948 [3], BPV has been widely applied in cardiovascular risk stratification [4]. It has also been shown to correlate with target organ damage [4]. Moreover, intraoperative BPV has been independently associated with a higher incidence of postoperative acute kidney injury (AKI) [5] and a 3.5-fold increase in 30-day mortality among patients undergoing noncardiac surgery [6,7]. Therefore, maintaining hemodynamic stability is a critical objective to reduce postoperative complications.

Despite its significance, high intraoperative blood pressure variability (HIBPV) is often overlooked and inadequately explored in clinical practice. The mechanisms underlying HIBPV are complex, involving interactions among baseline vascular function, autonomic nervous system activity, types and doses of anesthetics, intraoperative blood loss, and fluid management [8–10]. Additionally, several studies have confirmed the association between BPV and adverse postoperative outcomes [4,7,11]. Notably, Wiórek et al. reported that elevated intraoperative BPV significantly increased postoperative mortality risk in patients undergoing noncardiac surgery [7]. Unfortunately, most existing studies employed traditional regression-based approaches and treated BPV as a predictor variable rather than a direct modeling target, lacking systematic identification and characterization of HIBPV [6,12].

Due to its strength in modeling high-dimensional and nonlinear clinical data, machine learning (ML) has gained increasing traction in medical research [11,13,14]. Importantly, ML techniques have been successfully applied to predict perioperative hemodynamic events. For instance, Chen and Zhang developed ML-based models using Extreme Gradient Boosting and RF to predict induction-phase intraoperative hypotension [14]. Hatib et al. also proposed the Hypotension Prediction Index, a proprietary ML algorithm trained on high-fidelity arterial waveform data [15]. However, its black-box nature limits transparency and clinical applicability. SHapley Additive Explanations (SHAP) and other explainable ML techniques have been incorporated to improve model transparency, enabling global and individualized interpretation of feature contributions and enhancing clinical applicability [16–18]. In this regard, Dai et al. applied SHAP-based explainable ML to intraoperative blood pressure time series to predict postoperative acute kidney injury in cardiac surgery patients [18]. Furthermore, Kouz et al. used unsupervised clustering to identify IOH phenotypes, providing mechanistic insights. However, their approach was not designed for supervised modeling [11].

Despite progress in ML applications for intraoperative hemodynamic disturbances, HIBPV remains unexplored mainly as a distinct modeling target. Additionally, its physiological drivers may differ substantially across age groups. Given physiological differences in autonomic regulation, vascular reactivity, and anesthetic sensitivity between pediatric, adult, and elderly patients, subgroup-based modeling of HIBPV is of both theoretical and clinical importance. Therefore, this study constructed interpretable supervised ML models to classify patients with HIBPV. Additionally, we examined how key perioperative factors contribute to HIBPV across different age groups. A comparative summary of existing modeling approaches, including representative algorithms, application domains, and methodological limitations, is presented in Table 1 to contextualize the methodological positioning and innovation of this study [6,7,11,14,15,18].

**Table 1.** Comparison of representative modeling approaches for perioperative blood pressure dynamics.

Study	Modeling focus	Method type	Explainability	Subgroup analysis	Key limitation	Contribution of present study
Wiórek et al.	BPV → mortality	Traditional regression	No	No	No predictive model; poor generalizability	Enables individualized BPV prediction
Maheshwari et al.	BPV + waveform → IOH	Regression + BPV	No	No	No real-time prediction	Combines waveform & demographics under ML
Chen & Zhang	Induction IOH prediction	ML (XGBoost, RF, etc.)	No	No	No BPV modeling or interpretability	Extends ML to BPV with interpretability
Hatib et al.	Real-time IOH prediction (HPI)	Proprietary ML (HPI)	No	No	Black-box model; lacks transparency	Provides transparent alternative to HPI
Kouz et al.	IOH phenotype clustering	Unsupervised clustering	No	No	Not predictive; pattern only	Translates phenotypes into predictions
Dai et al.	Time-series BP → AKI (TA-AAD)	Explainable ML (XGBoost + SHAP)	Yes	No	Cardiac surgery specific; no BPV index	Applies explainable ML beyond cardiac field
Present Study	HIBPV detection	Explainable ML (XGBoost + SHAP)	Yes	Yes	—	First interpretable HIBPV model with subgrouping

BPV: blood pressure variability; HIBPV: high intraoperative blood pressure variability; IOH: intraoperative hypotension; ML: machine learning; RF: random forest; XGBoost: extreme gradient boosting; SHAP: Shapley additive explanations; HPI: Hypotension Prediction Index; TFT: temporal fusion transformer; ASA: American Society of Anesthesiologists; AKI: acute kidney injury; TA-AAD: type A acute aortic dissection.

## 2. Materials and methods

### 2.1. Study design

This study employed a retrospective cohort design, collecting data from general anesthesia surgeries conducted at Beijing Tsinghua Changgung Hospital between March 2016 and April 2022. The study was approved by the Ethics Committee of Beijing Tsinghua Changgung Hospital (Approval No. 22232-4-02), was exempted from the requirement for informed consent due to the use of de-identified retrospective data, and was conducted in accordance with the ethical principles outlined in the Declaration of Helsinki. Additionally, it was registered on ClinicalTrials.gov (Registration No: NCT05698433). Patient data were obtained through systematic queries of the Hospital Information System (HIS) and Anesthesia Information Management System (AIMS). Detailed entries of the original data types are provided in Supplementary Material 1.

The inclusion criteria for the study were as follows: (1) the patient must undergo noncardiac surgery, (2) intraoperative mean arterial pressure (MAP) measurements should be recorded at 5-minute intervals, (3) the patient must have an ASA classification I to V, and (4) the patient’s age must be between 1 and 99 years.

The exclusion criteria included: (1) use of local anesthesia, (2) non-intubated general anesthesia, (3) missing more than four consecutive MAP measurements (equivalent to a 20-minute gap), (4) an intraoperative maximum mean MAP above 300 mmHg, (5) an intraoperative minimum MAP below 20 mmHg, and (6) the absence of intraoperative arterial blood gas (ABG) results. Cases with missing data or unclear information were excluded to ensure dataset completeness and accuracy. The requirement for individual informed consent was waived, given the retrospective nature of this study. We followed the STROBE guidelines to optimize the reporting and methodological design, ensuring the study’s transparency, completeness, and reproducibility.

### 2.2. Data collection

Patient baseline information was extracted from the HIS, including height, weight, gender, and preoperative disease diagnoses. Intraoperative data, including vital signs monitoring, medication usage, fluid management, ABG, and surgery types, were obtained from the AIMS. Medications with an intraoperative usage frequency exceeding 10% were selected to ensure adequate representativeness and relevance of the analysis. The selected medications included sevoflurane, propofol, dexmedetomidine, midazolam, remifentanil, sufentanil, methoxamine, and rocuronium. To analyze the potential impact of preoperative diagnoses on HIBPV, we included diagnostic variables related to intraoperative circulatory fluctuations, such as heart failure, renal failure, and hypertension. During the data cleaning process, we removed cases that lacked intraoperative blood pressure data or other critical perioperative information. Additionally, we addressed outliers and ambiguous samples. Variables with over 5% missing data were excluded, while those with 5% or less missing data underwent mean imputation (Supplementary Material 1). Notably, this method guaranteed that the management of absent data did not substantially modify the overall data structure while preserving the dataset’s integrity.

### 2.3. Definition for HIBPV

The study only includes HIBPV as a categorical outcome measure. Intraoperative BPV has been the subject of several critical studies in recent years [4,6,7]. Thus, this study uses a BPV calculation method based on the coefficient of variation (CV), quantifying BPV by calculating the CV of the MAP intraoperatively—the ratio of the standard deviation of MAP to the mean MAP. This calculation method considers the patient’s blood pressure fluctuations and baseline blood pressure levels, effectively reflecting the impact of baseline blood pressure on BPV [12]. Moreover, the validity of this definition has been confirmed in multiple previous studies [12,19,20]. In this study, cases where the CV of MAP exceeds 20% are defined as HIBPV cases [21]. During the study, we collected blood pressure data at 5-minute intervals following the initiation of anesthesia. Invasive blood pressure values were taken for patients who underwent arterial catheterization. If the invasive blood pressure was not recorded, a noninvasive blood pressure cuff was used to measure it. Only one technique of blood pressure recording was standardized for each procedure.

### 2.4. Statistical analysis

This study employed four different classification models: Extreme Gradient Boosting (XGBoost) [22], Light Gradient Boosting Machine (LightGBM) [23], Random Forest (RF) [24], and Logistic Regression (LR) [25]. XGBoost is an efficient algorithm that improves identification accuracy by constructing multiple decision trees and utilizing Gradient Boosting techniques [22]. It is particularly well-suited for large and feature-rich datasets. LightGBM, a lightweight version of XGBoost, enhances computational efficiency and memory usage, making it perform exceptionally well with larger datasets [23]. RF is an ensemble learning algorithm that boosts overall performance by generating multiple decision trees and combining their pattern recognition results [24]. Lastly, LR, a traditional statistical method, remains effective when the relationships between features are linear [25].

**Figure 1.**
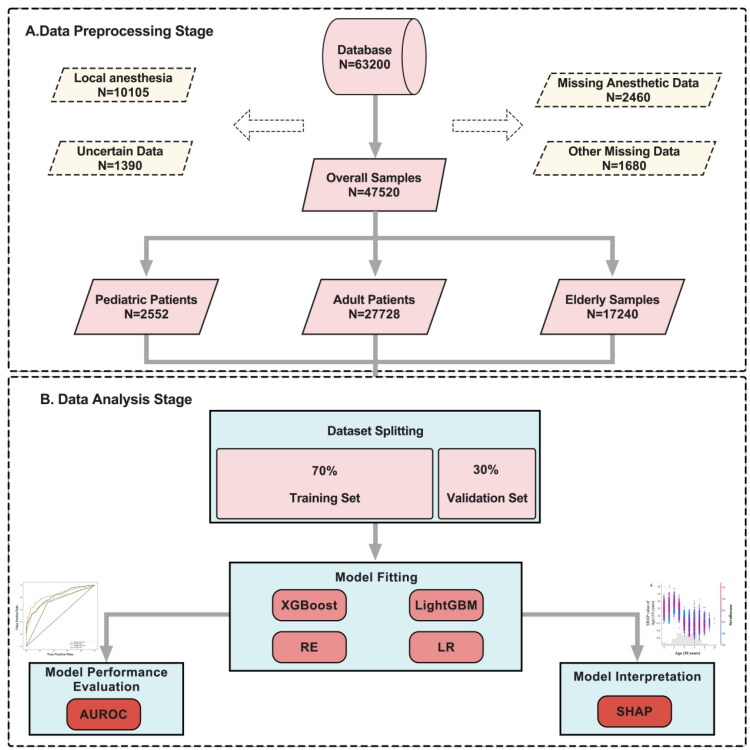
The process of constructing the interpretable model in pattern recognition of high intraoperative blood pressure variability in noncardiac surgery; XGBoost, extreme gradient boosting;RF, random Forest; LGBoost, light Gradient Boosting; LR, logistic regression.

The final performance evaluation was primarily based on the area under the Receiver Operating Characteristic curve (AUROC). We also considered additional metrics to assess the model’s effectiveness across the overall population, including accuracy, specificity, and F1 score. To determine the model’s effectiveness in various age subgroups (Figure 1), we computed the AUROC using four distinct ML algorithms in pediatric (under 18 years old), adult (between 18 and 65 years old), and elderly patients (65 years old and above). We adopted a bootstrap method for calculating each algorithm’s 95% CIs of the AUROC scores across different patient subgroups. This method, involving 2,000 resamples of the original dataset, enhances the accuracy and reliability of our performance metrics. A complete list of the 52 perioperative variables used across all four machine learning (ML) models is provided in Supplementary Table 1.

The SHAP (SHapley Additive exPlanations) method [26], a leading model explanation tool, utilizes SHapley values from game theory to quantify each feature’s contribution to the model’s identification results. Notably, this method can reveal how features influence the model’s decision-making process and provide clear and detailed explanations. The explanatory analysis was primarily based on the XGBoost model, chosen for its high compatibility with the SHAP method [26]. Two primary SHAP explanation methods were used: global and interactive interpretability methods. The global interpretability method assesses each feature’s overall influence and direction on the model’s identification results across the entire dataset and within specific subgroups [27]. In contrast, the interactive interpretability method examines the interactions between features, highlighting that their effects do not occur independently but rather interact with one another during the pattern recognition process [28]. Our study offers a comprehensive analysis of how sevoflurane dosage interacts with the patient’s age, blood calcium levels, and hypertension diagnosis to affect HIBPV. Combining these two methodologies gives valuable insights into the model’s decision-making process, significantly improving interpretability [29]. The model was developed using Python 3.9, with data processing performed using pandas 1.3.4, model training and parameter optimization utilizing Scikit-Learn 0.24.2, and interpretability analysis conducted with SHAP 0.41.0.

## 3. Results

From an initial dataset of 63,200 surgical cases (Figure 1), we excluded 10,105 cases with local anesthesia, 2,460 cases lacking intraoperative BP data, 1,680 cases with missing ABG records, and 1,390 cases with anomalous ABG values, resulting in 47,520 cases for final analysis. Among these, 1,996 surgeries (4.2%) exhibited HIBPV. The cohort-level distribution of MAP variability demonstrated that 79.0% of patients exhibited variability between 10 ∼ 20%, 16.8% had variability <10%, and 4.2% exceeded 20%. The highest degrees of variability were observed in vascular and neurosurgical procedures, whereas obstetrical and urological surgeries showed the lowest levels (Supplementary Figures S1 and S2). The preliminary model incorporated 52 perioperative features (Table 2). Patients had a mean age of 43.3 years, height of 162.5 cm, and weight of 64.2 kg; 45.6% were male. The mean surgical duration was 2.3 h. Crystalloid administration exceeded colloid use; average urine output and blood loss were 324.8 and 82.4 mL, respectively. Gastrointestinal surgeries under general anesthesia were most common (7,011 cases, 15%), whereas vascular surgeries were the least frequent (813 cases, 1.7%). ABG values remained within normal physiological electrolyte ranges (Table 2).

**Figure 2.**
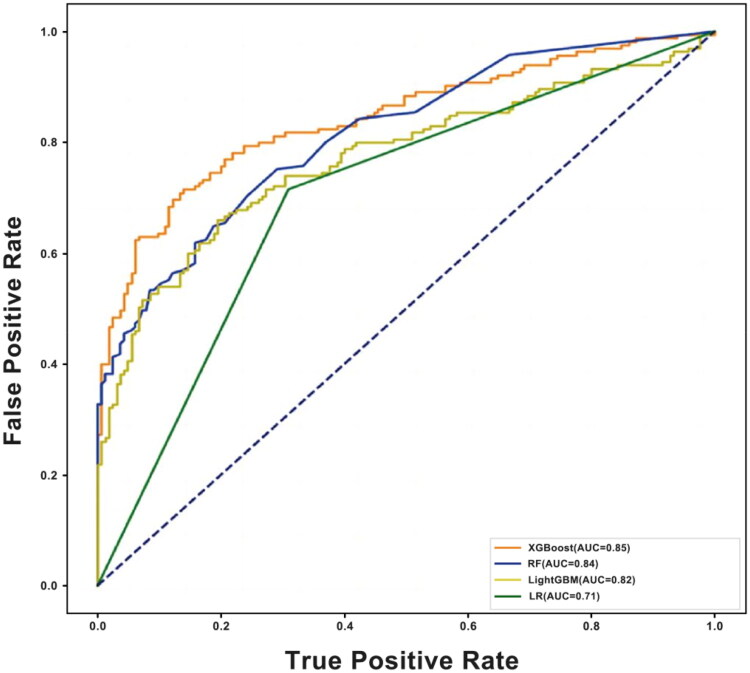
Receiver operating characteristic (ROC) curves of four machine learning models for predicting high intraoperative blood pressure variability (HIBPV) during noncardiac surgery. The area under the curve (AUC) was 0.85 (95% CI: 0.84–0.86) for XGBoost, 0.84 (0.82–0.85) for random Forest (RF), 0.82 (0.80–0.84) for light Gradient Boosting Machine (LightGBM), and 0.71 (0.69–0.74) for logistic regression (LR). These results demonstrate the superior discriminative performance of tree-based ensemble methods in identifying HIBPV patterns; XGBoost, extreme gradient boosting: RF, random Forest; LGBoost, light Gradient Boosting; LR, logistic regression.

**Table 2.** Baseline and intraoperative characteristics of the study population.

Perioperative features	Unit	Value(mean ± SD)	Perioperative features	Unit	Value(mean ± SD)
**Baseline Information**			SD of MAP	mmHg	10.97 ± 9.97
Age	(years)	43.33 ± 18.83	Average MAP	mmHg	83.82 ± 4.34
Height	(cm)	162.48 ± 17.52	**Arterial Blood Gases**		
Weight	(kg)	64.19 ± 24.51	pH	–	7.38 ± 0.13
Male	(%)	45.56	Hb	(g/dL)	12.03 ± 2.84
Baseline SBP	mmHg	124.04 ± 28.25	PaCO_2_	(mmHg)	39.37 ± 5.35
Baseline DBP	mmHg	72.11 ± 17.38	PaO_2_	(mmHg)	139.73 ± 94.07
**Preoperative diagnosis**			HCO₃⁻	(mmol/L)	23.94 ± 2.59
Hypertension	(%)	25.38	K⁺	(mmol/L)	3.55 ± 0.52
Cardiac insufficiency	(%)	5.31	Ca²⁺	(mmol/L)	1.11 ± 0.06
Renal insufficiency	(%)	1.95	Na⁺	(mmol/L)	137.31 ± 6.72
**Surgical Classification**			SO_2_	(%)	98.21 ± 1.50
Gynecological	(%)	13.86	Lactate	(mmol/L)	1.24 ± 0.74
Hepatic	(%)	2.61	AG	(mmol/L)	7.85 ± 1.01
Orthopedic	(%)	12.95	BE	(mmol/L)	−0.84 ± 3.03
General	(%)	12.66	**Intraoperative Drug**		
Urological	(%)	8.21	Sevoflurane	(%)	2.78 ± 1.70
Neurosurgical	(%)	4.86	Propofol	(mg)	172.79 ± 224.92
Nephrotic	(%)	9.81	Dexmedetomidine	(ug)	58.54 ± 90.01
Gastrointestinal	(%)	14.99	Midazolam	(mg)	0.95 ± 0.91
Thoracic	(%)	2.94	Remifentanil	(ug)	720.45 ± 821.51
Vascular	(%)	1.71	Sufentanil	(ug)	28.27 ± 26.92
Otolaryngologic	(%)	10.97	Methoxamine	(mg)	1.68 ± 7.28
**Fluid Balance**			Rocuronium	(mg)	37.8 ± 41.92
Crystalloid solution	(ml)	1214.44 ± 980.14	Dexamethasone	(mg)	1.97 ± 3.82
Colloidal solution	(ml)	247.25 ± 438.49	Atropine	(mg)	0.26 ± 0.38
Urine Output	(ml)	324.81 ± 275.35	Ephedrine	(mg)	2.43 ± 3.34
Blood loss	(ml)	82.41 ± 320.20	Noradrenaline	(ug)	714.99 ± 163.51
**Intraoperative vital signs**			Furosemide	(mg)	1.81 ± 6.52
Average HR	beats/min	73.29 ± 12.25	**Operation Duration**		
Average BIS	–	47.83 ± 7.91	Duration	(hours)	2.31 ± 2.60

SBP: Systolic blood pressure; DBP: Diastolic blood pressure; HR: Heart rate; pH: Potential of hydrogen; BIS: Bispectral Index; MAP: mean arterial pressure; THbc: Hemoglobin; PaCO_2_: Partial pressure of arterial carbon dioxide**;** PaO_2_: Partial pressure of arterial oxygen**;** HCO₃⁻: Bicarbonate ion; K⁺: Potassium ion; Ca²⁺: Calcium ion; Na: Sodium ion; SO_2_: Oxygen saturation**;** AG: Anion gap**;** BE: Base excess.

The models included 70% of the samples as the training set and 30% as the test set. Based on the training set, a stratified k-fold cross-validation (*k* = 5) strategy was employed. The ROC curve analysis of the test set (Figure 2) shows that the XGBoost and the RF algorithms exhibit the best performance. Notably, the test results (Table 3) indicated diverse performance levels among various models within the overall population. Furthermore, XGBoost and RF demonstrated superior performance, with XGBoost attaining an AUROC of 0.85 (95% CI: [0.84, 0.86]) and RF achieving 0.84 (95% CI: [0.82, 0.85]). The LightGBM model exhibited strong performance, achieving an AUROC of 0.82 (95% CI: [0.80, 0.83]). The LR model showed a lower AUROC of 0.71 (95% CI: [0.69, 0.72]). The XGBoost algorithm consistently achieves optimal discrimination across all subgroups within the population, with a stable AUROC value above 0.8 (Supplementary Table 2).

**Table 3.** Comparison of classification performance across machine learning models for HIBPV prediction.

	AUROC	AUC lower	AUC upper	Accuracy	Precision	Sensitivity	Specificity	F1-score
XGBoost	0.85	0.84	0.86	0.77	0.79	0.79	0.75	0.77
RF	0.84	0.82	0.85	0.75	0.74	0.76	0.74	0.75
LightGBM	0.82	0.80	0.83	0.74	0.74	0.76	0.73	0.75
LR	0.71	0.69	0.72	0.71	0.71	0.72	0.71	0.71

AUROC: Area Under the Receiver Operating Characteristic Curve; F1-score: balanced F Score**;** XGBoost: extreme gradient boosting**;** RF: random forest**;** LGBoost: Light Gradient Boosting**;** LR: logistic regression.

**Figure 3.**
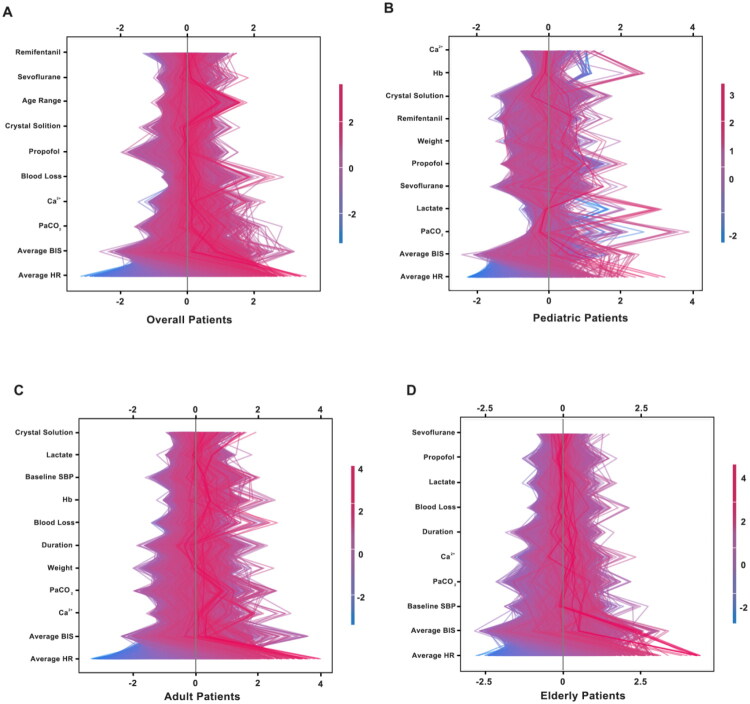
The top 10 significant features for identifying HIBPV in noncardiac surgery. (A): overall patients: the SHAP values and feature rankings are for the entire patient cohort, providing a general overview of the significant features and their impacts. (B): pediatric patients (under 18 years old): here, the SHAP values and rankings are specific to pediatric patients. The data reflects the unique characteristics and relationships of these features within this age group.(C): adult patients (between 18 and 65 years old): the SHAP values and feature rankings for adult patients are shown. The differences in values and rankings compared to other populations may be due to the physiological and surgical differences in adults. (D): elderly patients (65 years old and above): this Sub-figure presents the SHAP values and feature rankings for the elderly patient population. The data helps to understand the importance of features in predicting HIBPV in this age group, considering their specific health conditions. The x-axis represents SHAP values, which convey the magnitude and direction of each feature’s contribution to the model’s prediction. Negative SHAP values (left of center) imply a decreased risk of HIBPV, and positive values (right of center) suggest an increased risk. The y-axis shows a ranked list of the ten most influential traits. The color of the lines corresponds to the feature values: red indicates an increased probability of HIBPV occurrence as the value rises, and blue indicates a decreased probability.

The SHAP analysis of the overall population (Figure 3) identified the key factors influencing HIBPV. The features were ranked based on their average absolute SHAP values, with those at the bottom having the greatest impact on the HIBPV. Red lines indicate an increase in the probability of HIBPV occurrence, while blue lines indicate a decrease in this probability. The two most significant factors influencing the HIBPV for the general population are the average HR during surgery (SHAP value range: −4 to 4) and the average BIS value during surgery (SHAP value range: −2 to 3.5). In the ABG analysis of the overall population (Figure 3A), PaCO_2_ and arterial blood calcium levels are significant determinants of intraoperative BPV. The SHAP value range for arterial PaCO_2_ is −1.5 to 2, whereas for blood calcium it is −1 to 2. The associations between PaCO_2_ (SHAP value range: −0.5 to 4) are more pronounced in pediatric patients (Figure 3B). For adults (Figure 3C), baseline SBP (SHAP range: −1.5 to 1.5) and intraoperative blood loss (SHAP range: −1.0 to 2.0) were predominant drivers. In the elderly (Figure 3D), average HR (SHAP range: −2.5 to 2.3) remained significant but attenuated compared to younger cohorts. Sevoflurane dosage (SHAP range: −1.5 to 1.2) demonstrated a protective effect, with higher doses reducing HIBPV risk.

**Figure 4.**
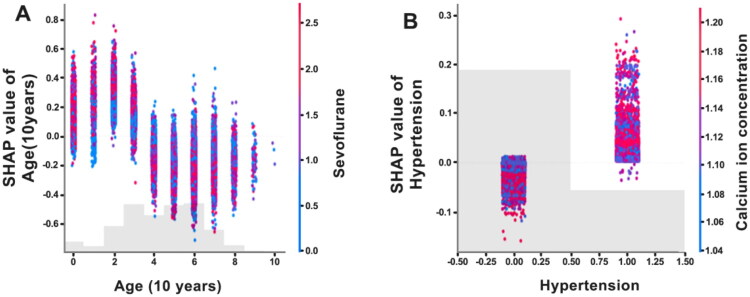
SHAP interactive explanation plot for all patients from the testing set of the XGBoost model. (A): interaction between the dose of sevoflurane and patient age on intraoperative blood pressure variability. (B): interaction between arterial blood calcium levels and the history of hypertension on intraoperative blood pressure variability. The SHAP value is displayed on the Y-axis, with a negative number indicating a beneficial impact on the forecast by lowering BPV, while a positive value indicates an adverse influence on the prediction by increasing blood pressure fluctuation. The X-axis coordinates indicate the variation in the value of the specified influencing factor, while the predictive factor on the right side of the chart represents the interacting component that interacts with the specified influencing factor. The color red symbolizes an augmentation in the value of the interaction component, whereas the color blue signifies a reduction in the value of the interaction factor.

The interactive interpretability analysis revealed the interactions among factors influencing HIBPV, particularly the effects of sevoflurane levels, patient age, arterial blood calcium, and the incidence of intraoperative HIBPV in patients with hypertension. As shown in Figure 4A, there is a significant interaction between sevoflurane usage and age. Specifically, for young patients aged 20 ∼ 30, an increase in sevoflurane usage may increase BPV (SHAP values up to 0.8). Conversely, for elderly patients aged 60 ∼ 70, an increase in sevoflurane usage appears to reduce BPV (SHAP values as low as −0.8). For middle-aged patients, the impact of sevoflurane usage on BPV is minimal. Figure 4B illustrates the interaction between arterial blood calcium levels and preoperative hypertension diagnosis. Increased intraoperative arterial blood calcium levels (greater than 1.10 mmol/L) reduce the probability of HIBPV in patients without hypertension (with SHAP values as low as −0.15). In contrast, it increases the likelihood of HIBPV in patients with hypertension (with SHAP values up to 0.25).

## 4. Discussion

This study demonstrates that supervised ML models can effectively classify HIBPV in a large cohort of noncardiac surgery patients. The models showed consistently strong performance across pediatric, adult, and elderly subgroups. Key features influencing HIBPV included the average HR during surgery, BIS, and the PaCO_2_. Furthermore, the analysis uncovered age-specific interactions related to sevoflurane dosage and a differential relationship between intraoperative blood calcium levels and preexisting hypertension, suggesting variability in physiological responses among different age groups.

Intraoperative BPV is known to be associated with an increased risk of postoperative complications [4,6,7]. Although anesthetic and vasoactive agents ty[ically help stabilize hemodynamics during surgery, identifying HIBPV remains challenging due to the complex interplay between baseline conditions and intraoperative dynamics. Specifically, certain intraoperative events may independently contribute to BPV, in addition to factors accounted for by baseline characteristics or surgical type. To address this, our study employed supervised ML models to classify patients with HIBPV directly, rather than predicting postoperative outcomes based on these variations.

Previous studies have successfully applied ML techniques to intraoperative risk prediction. Hatib et al. used arterial waveform data to forecast hypotension with high sensitivity and specificity [15], while Jalali et al. predicted transfusion requirements with an AUROC up to 0.91 [30]. These findings support the applicability of supervised ML in perioperative hemodynamic modeling, which aligns with our use of pattern recognition to characterize HIBPV.

XGBoost and RF demonstrated the strongest ability to identify HIBPV based on perioperative data among the evaluated ML algorithms. As illustrated in Figure 2, their ROC curves showed visually comparable performance. Specifically, the AUROC of XGBoost was 0.85 (95% CI: 0.84–0.86), and RF achieved 0.84 (95% CI: 0.82–0.85). These AUROC values aligned well with other performance metrics, reinforcing their robustness in HIBPV identification. Although all models were trained on the same input features, the LR model showed relatively lower predictive performance. This is likely due to its limited capacity to model nonlinear relationships and feature interactions compared to tree-based ensemble methods.

The extent to which a model’s interpretability aligns with clinical reasoning significantly affects its credibility in practice. As illustrated in Figure 3, higher intraoperative HR is associated with increased HIBPV risk. This relationship likely indicates inadequate analgesia that triggers sympathetic activation and hemodynamic instability [31]. Additionally, variations in BIS, which serves as a measure of sedation depth, have also been linked to increased BP variability. Both under- and over-sedation can destabilize vascular tone, consistent with prior clinical observations [32]. PaCO_2_ was another significant contributor to BP variability, as elevated levels induce vasodilation and may affect cardiac output and HR [33]. To assess cross-model consistency, we compared the top ten features from each algorithm. Average HR, BIS, PaCO_2_, Ca^2+^, blood loss, and crystalloid volume were consistently identified as top predictors. This discovery validates the selected variables’ dependability and improves our results’ clinical interpretation.

PaCO_2_ is recognized as a highly influential variable in pediatric patients (Figure 3B). This is likely due to the underdeveloped central chemoreceptors and immature autonomic regulation in children, which limit their capacity to compensate for CO_2_ retention. Even minor changes in PaCO_2_ under general anesthesia can trigger the sympathetic nervous system, leading to significant hemodynamic disturbances [34]. This heightened physiological sensitivity makes pediatric patients susceptible to circulatory instability, which can result from respiratory acidosis or hyperventilation [34]. In adult patients (Figure 3C), baseline SBP and intraoperative blood loss have been identified as principal risk factors. Patients with a history of hypertension tend to have reduced vascular compliance and elevated autoregulatory thresholds, making them more susceptible to significant blood pressure fluctuations during anesthetic induction [35]. In addition, hypovolemia caused by surgical hemorrhage may compromise circulatory compensation, triggering reflex tachycardia or tissue hypoperfusion, thereby intensifying BPV [36,37]. These subgroup-specific SHAP insights support the physiological interpretability of the model and its alignment with clinical expectations. In elderly patients (Figure 3D), although average heart rate (HR) remained a key predictor, its SHAP contribution was slightly attenuated. Notably, sevoflurane exhibited a dose-dependent protective trend [38]. Elderly individuals are characterized by diminished vascular compliance, impaired autonomic reflexes, and blunted sympathetic responsiveness [39,40]. Sevoflurane may mitigate hemodynamic stress through sympathetic inhibition, small-artery vasodilation, and improved coronary perfusion, thereby offering a plausible mechanism for its stabilizing role in elderly patients.

Interestingly, vasoactive medications were not among the top discriminative features of our models that contributed to HIBPV classification. This finding likely reflects the real-time titration of these agents in response to dynamic blood pressure changes. Instead, baseline physiological characteristics—such as internal milieu and preoperative vital signs—appeared to influence intraoperative BPV more. These results suggest complex interactions between pharmacologic interventions and individual patient characteristics in maintaining intraoperative circulatory stability.

Figure 4A illustrates a significant interaction between sevoflurane dosage and patient age, indicating that its influence on BPV differs across age groups. In younger patients (aged 20 ∼ 30), higher sevoflurane levels were associated with increased BPV, whereas in older adults (aged 60 ∼ 70), the same exposure appeared to reduce variability. Although research on the elderly is relatively limited, existing evidence indicates that sevoflurane is generally well tolerated in this population. It has been shown to promote coronary vasodilation, enhance myocardial perfusion, and reduce vascular resistance, thereby improving cardiac function and lowering arrhythmia risk [41]. These properties support its preferential use in older adults [42]. Nonetheless, further investigation is warranted to elucidate the age-dependent effects of sevoflurane on intraoperative hemodynamics.

Figure 4B illustrates a significant interaction between intraoperative arterial calcium levels and preexisting hypertension. In normotensive patients, higher calcium levels appeared to be associated with a reduced HIBPV risk, while in hypertensive patients, elevated calcium was linked to an increased risk. These findings underscore the clinical relevance of perioperative calcium management, particularly in hypertensive individuals. Elevated intraoperative calcium may indicate underlying dysregulation, and transient hypercalcemia could predispose to coagulopathy or vascular reactivity, potentially exacerbating hypertensive responses [43]. Furthermore, hypercalcemia may promote vascular and coronary calcification, accelerate atherosclerosis, and contribute to hemodynamic instability [43].

This study has several limitations. First, it was conducted at a single center, which means that institution-specific surgical practices and anesthetic protocols may have influenced the circulatory dynamics, potentially restricting the applicability of the findings to other settings. Second, intraoperative BPV can vary significantly across different surgeries; however, this procedural heterogeneity was not fully captured or stratified in the current model. This oversight may have affected the model’s ability to discriminate effectively in specific surgical contexts. Lastly, although the interpretability techniques can uncover salient feature associations, the conclusions drawn remain correlational. Further research is needed using prospective designs or causal inference frameworks to establish causal relationships and understand the mediating mechanisms. Future studies should aim for multi-center, cross-procedure designs and adopt advanced statistical or ML techniques to enhance the generalizability and clinical relevance of the findings.

## 5. Conclusion

This study utilized ML algorithms to examine patients’ baseline information and intraoperative characteristics across the overall population and various subgroups in a large cohort dataset. Both XGBoost and RF algorithms exhibited robust and comparable performance in identifying HIBPV. Intraoperative average HR and BIS are recognized as significant indicators of HIBPV risk. The interaction between sevoflurane dosage and age, as well as elevated arterial blood calcium levels during general anesthesia in patients diagnosed with hypertension, represents additional risk factors. These models provide practical tools and potential interventions for the perioperative management of high-risk HIBPV patients.

## Supplementary Material

Supplemental Material

## Data Availability

The data for this retrospective cohort study at Beijing Tsinghua Changgung Hospital cannot be publicly shared due to ethical, privacy, and institutional data policy. Sufficient details on data collection methods, including patient selection criteria and variables, are provided.
