# Supplementary material for: Identifying patterns of high intraoperative blood pressure variability in noncardiac surgery using explainable machine learning: a retrospective cohort study
Source: Ann Med. 2025 Jul 24;57(1):2537920. doi: 10.1080/07853890.2025.2537920 (PMC12291218; doi:10.1080/07853890.2025.2537920)
Supplement: Supplemental Material [file IANN_A_2537920_SM3678.zip › suppl_data/IANN-2024-6094.R1-Supplementary_Table_S1-Clean.docx]

**Supplementary Table S1. Full list of 52 perioperative features used in the development of all machine learning models**

Part A – Shared Demographics, Diagnoses, Vitals, Surgery, Fluids

| XGBoost features | Random Forest features | LightGBM features | Logistic Regression features |
| --- | --- | --- | --- |
| Age | Age | Age | Age |
| Height | Height | Height | Height |
| Weight | Weight | Weight | Weight |
| Male | Male | Male | Male |
| Baseline SBP | Baseline SBP | Baseline SBP | Baseline SBP |
| Baseline DBP | Baseline DBP | Baseline DBP | Baseline DBP |
| Hypertension | Hypertension | Hypertension | Hypertension |
| Cardiac insufficiency | Cardiac insufficiency | Cardiac insufficiency | Cardiac insufficiency |
| Renal insufficiency | Renal insufficiency | Renal insufficiency | Renal insufficiency |
| Gynecological | Gynecological | Gynecological | Gynecological |
| Hepatic | Hepatic | Hepatic | Hepatic |
| Orthopedic | Orthopedic | Orthopedic | Orthopedic |
| General | General | General | General |
| Urological | Urological | Urological | Urological |
| Neurosurgical | Neurosurgical | Neurosurgical | Neurosurgical |
| Nephrotic | Nephrotic | Nephrotic | Nephrotic |
| Gastrointestinal | Gastrointestinal | Gastrointestinal | Gastrointestinal |
| Thoracic | Thoracic | Thoracic | Thoracic |
| Vascular | Vascular | Vascular | Vascular |
| Otolaryngologic | Otolaryngologic | Otolaryngologic | Otolaryngologic |
| Crystalloid solution | Crystalloid solution | Crystalloid solution | Crystalloid solution |
| Colloidal solution | Colloidal solution | Colloidal solution | Colloidal solution |
| Urine Output | Urine Output | Urine Output | Urine Output |
| Blood loss | Blood loss | Blood loss | Blood loss |
| Average HR | Average HR | Average HR | Average HR |
| Average BIS | Average BIS | Average BIS | Average BIS |

Part B – Shared ABG and Medication Features

| XGBoost features | Random Forest features | LightGBM features | Logistic Regression features |
| --- | --- | --- | --- |
| pH | pH | pH | pH |
| Hb | Hb | Hb | Hb |
| PaCO_2_ | PaCO_2_ | PaCO_2_ | PaCO_2_ |
| PaO_2_ | PaO_2_ | PaO_2_ | PaO_2_ |
| HCO₃⁻ | HCO₃⁻ | HCO₃⁻ | HCO₃⁻ |
| K⁺ | K⁺ | K⁺ | K⁺ |
| Ca²⁺ | Ca²⁺ | Ca²⁺ | Ca²⁺ |
| Na⁺ | Na⁺ | Na⁺ | Na⁺ |
| SO_2_ | SO_2_ | SO_2_ | SO_2_ |
| Lactate | Lactate | Lactate | Lactate |
| AG | AG | AG | AG |
| BE | BE | BE | BE |
| Sevoflurane | Sevoflurane | Sevoflurane | Sevoflurane |
| Propofol | Propofol | Propofol | Propofol |
| Dexmedetomidine | Dexmedetomidine | Dexmedetomidine | Dexmedetomidine |
| Midazolam | Midazolam | Midazolam | Midazolam |
| Remifentanil | Remifentanil | Remifentanil | Remifentanil |
| Sufentanil | Sufentanil | Sufentanil | Sufentanil |
| Methoxamine | Methoxamine | Methoxamine | Methoxamine |
| Rocuronium | Rocuronium | Rocuronium | Rocuronium |
| Dexamethasone | Dexamethasone | Dexamethasone | Dexamethasone |
| Atropine | Atropine | Atropine | Atropine |
| Ephedrine | Ephedrine | Ephedrine | Ephedrine |
| Noradrenaline | Noradrenaline | Noradrenaline | Noradrenaline |
| Furosemide | Furosemide | Furosemide | Furosemide |
| Duration | Duration | Duration | Duration |

This table provides a comprehensive list of 52 perioperative variables that were uniformly applied as input features for the development of all machine learning models in this study, including XGBoost, Random Forest, LightGBM, and Logistic Regression. No model-specific feature selection was performed. To improve clarity, the feature list is divided into two parts: **Part A** includes baseline demographics, preoperative diagnoses, surgical classifications, intraoperative vital signs, and fluid balance parameters; **Part B** includes arterial blood gas values and intraoperative medications. Feature selection was based on clinical relevance and data availability. All variables were treated as structured, static inputs without temporal encoding, and no dimensionality reduction was applied prior to model training.

**Abbreviations:** SBP, systolic blood pressure; DBP, diastolic blood pressure; MAP, mean arterial pressure; HR, heart rate; BIS, bispectral index; Hb, hemoglobin; PaCO₂, partial pressure of arterial carbon dioxide; PaO₂, partial pressure of arterial oxygen; HCO₃⁻, bicarbonate ion; K⁺, potassium ion; Ca²⁺, calcium ion; Na⁺, sodium ion; SO₂, oxygen saturation; AG, anion gap; BE, base excess.
