# Supplementary material for: Identifying patterns of high intraoperative blood pressure variability in noncardiac surgery using explainable machine learning: a retrospective cohort study
Source: Ann Med. 2025 Jul 24;57(1):2537920. doi: 10.1080/07853890.2025.2537920 (PMC12291218; doi:10.1080/07853890.2025.2537920)
Supplement: Supplemental Material [file IANN_A_2537920_SM3678.zip › suppl_data/IANN-2024-6094.R1-Supplementary_Table_S2-Clean.docx]

Supplementary Table S2. Discriminative performance of four machine learning models for predicting high intraoperative blood pressure variability across age subgroups

| **Population Subgroup** | **Algorithm** | **AUROC** | **95% CI** |
| --- | --- | --- | --- |
| Overall Patients ( N = 47520 ) | XGBoost | 0.85 | [0.84 - 0.86] |
|  | RF | 0.84 | [0.82 - 0.85] |
|  | LightGBM | 0.82 | [0.80 - 0.83] |
|  | LR | 0.71 | [0.69 - 0.72] |
| Elderly Patients ( N = 17240 ) | XGBoost | 0.87 | [0.85 - 0.89] |
|  | RF | 0.85 | [0.83 - 0.87] |
|  | LightGBM | 0.83 | [0.81 - 0.85] |
|  | LR | 0.73 | [0.71 - 0.75] |
| Adult Patients ( N = 27728 ) | XGBoost | 0.84 | [0.82 - 0.85] |
|  | RF | 0.82 | [0.81 - 0.83] |
|  | LightGBM | 0.81 | [0.79 - 0.82] |
|  | LR | 0.69 | [0.68 - 0.70] |
| Pediatric Patients ( N = 2552 ) | XGBoost | 0.81 | [0.78 - 0.83] |
|  | RF | 0.78 | [0.75 - 0.80] |
|  | LightGBM | 0.75 | [0.73 - 0.77] |
|  | LR | 0.65 | [0.62 - 0.67] |

**Abbreviations:** AUROC, Area Under the Receiver Operating Characteristic Curve**;** CI, Confidence Interval; XGBoost, extreme gradient boosting**;** RF, random forest**;** LightGBM, Light Gradient Boosting Machine**;** LR, logistic regression
