# Supplementary material for: Identifying patterns of high intraoperative blood pressure variability in noncardiac surgery using explainable machine learning: a retrospective cohort study
Source: Ann Med. 2025 Jul 24;57(1):2537920. doi: 10.1080/07853890.2025.2537920 (PMC12291218; doi:10.1080/07853890.2025.2537920)
Supplement: Supplemental Material [file IANN_A_2537920_SM3678.zip › suppl_data/Supplementary Figure S1.docx]

 Supplementary Figure S1


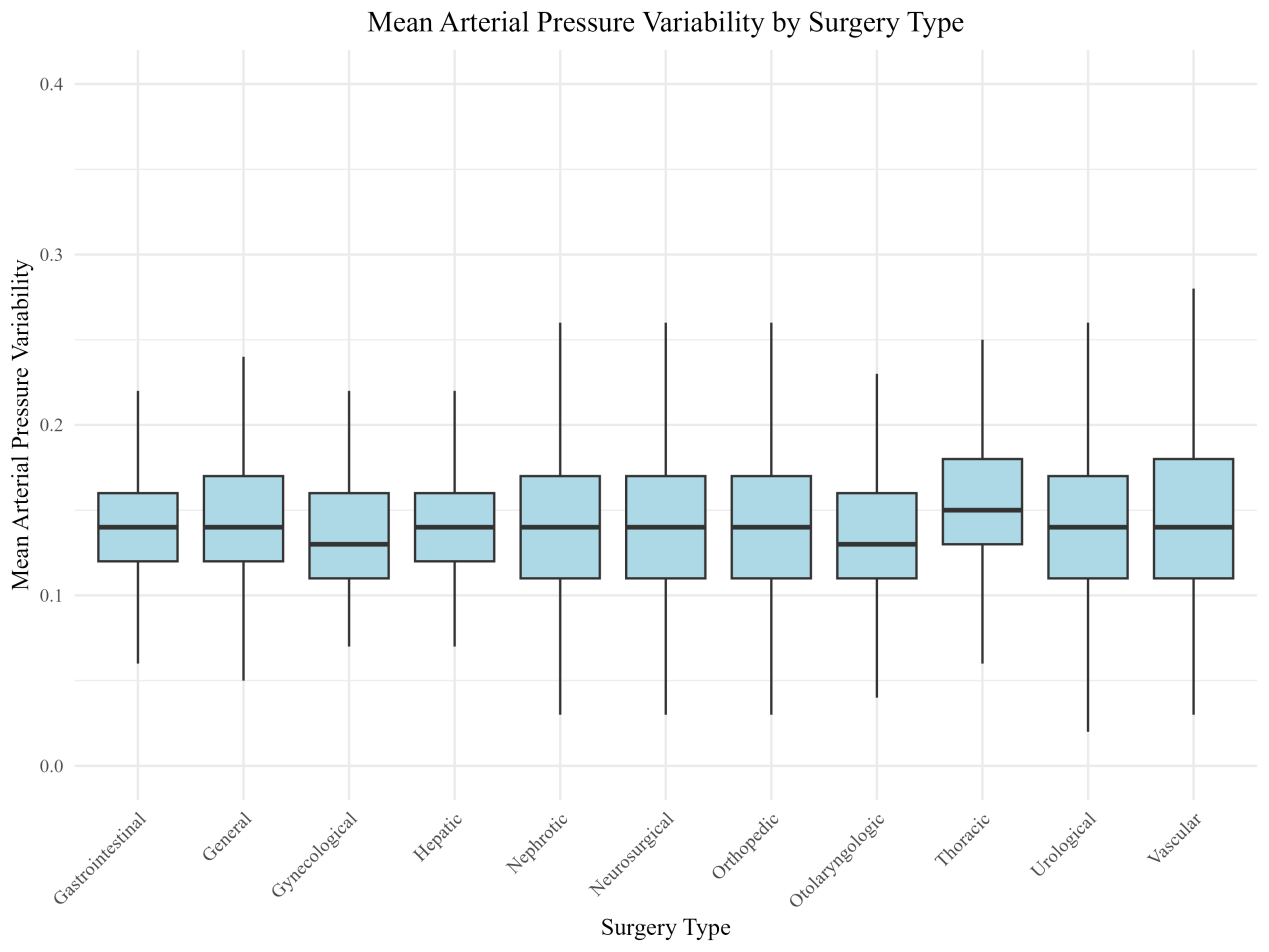


Mean Arterial Pressure Variability by Surgery Type：Box - and - whisker plot of mean arterial pressure variability across different types of non - cardiac surgeries. The x - axis represents the surgical types, including Gastrointestinal, General, Gynecological, Hepatic, Nephric, Neurosurgical, Orthopedic, Obstetrical, Thoracic, Urological, and Vascular surgeries. The y - axis represents the mean arterial pressure variability.
