# Supplementary material for: Identifying patterns of high intraoperative blood pressure variability in noncardiac surgery using explainable machine learning: a retrospective cohort study
Source: Ann Med. 2025 Jul 24;57(1):2537920. doi: 10.1080/07853890.2025.2537920 (PMC12291218; doi:10.1080/07853890.2025.2537920)
Supplement: Supplemental Material [file IANN_A_2537920_SM3678.zip › suppl_data/Supplementary Figure S2.docx]

Supplementary Figure S2


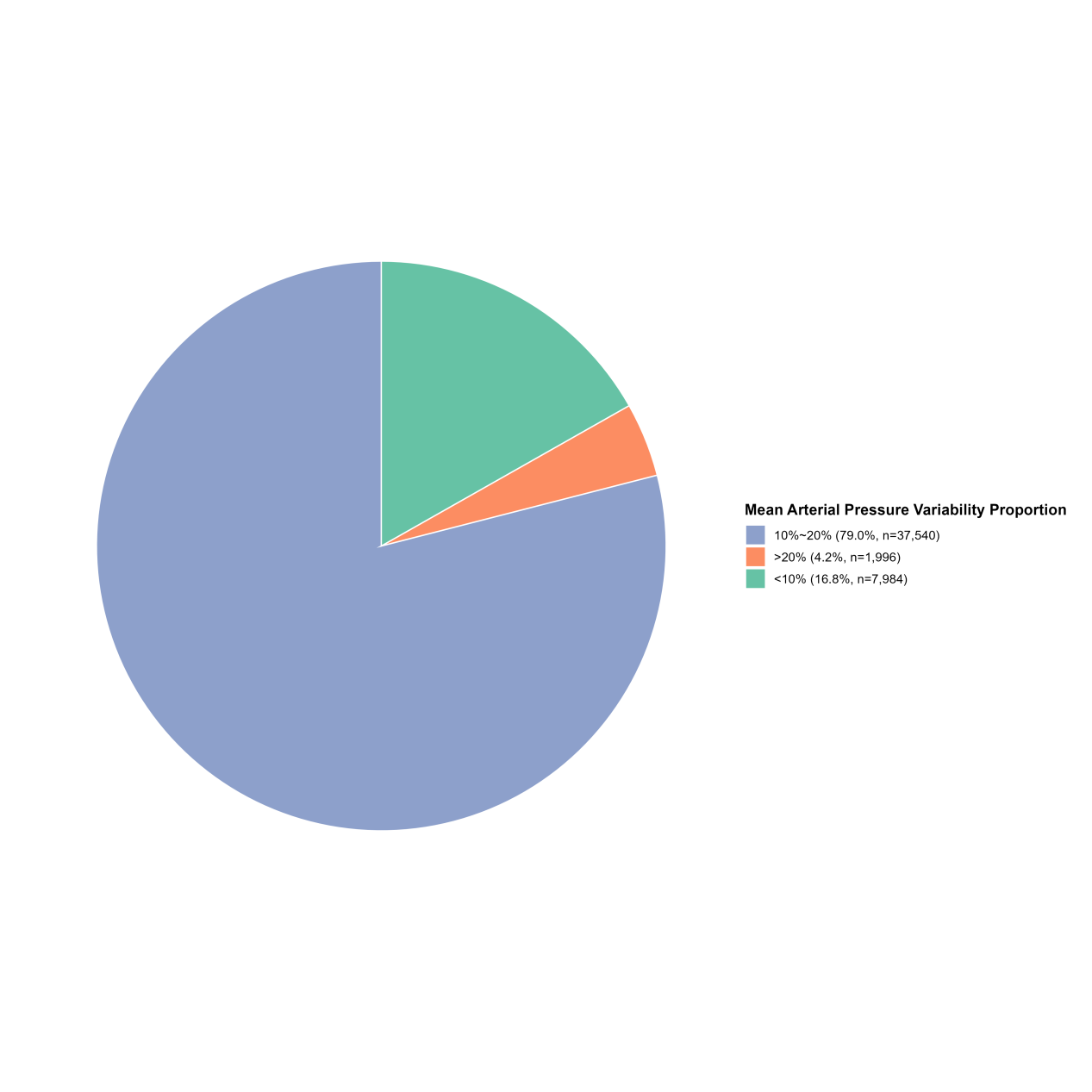


Figure caption: Pie - chart of the proportion of mean arterial pressure variability. The legend indicates three categories: 10% - 20% (accounting for 79.0%, n = 37,540), > 20% (accounting for 4.2%, n = 1,996), and < 10% (accounting for 16.8%, n = 7,9
