# Supplementary material for: Identifying patterns of high intraoperative blood pressure variability in noncardiac surgery using explainable machine learning: a retrospective cohort study
Source: Ann Med. 2025 Jul 24;57(1):2537920. doi: 10.1080/07853890.2025.2537920 (PMC12291218; doi:10.1080/07853890.2025.2537920)
Supplement: Supplemental Material [file IANN_A_2537920_SM3678.zip › suppl_data/Supplementary Material 1.docx]

**Supplementary Material : Hospital Database Management Procedures**

This retrospective cohort study is based on data from 47,520 general anesthesia surgeries performed in the central operating room at Beijing Tsinghua Changgung Hospital between March 2016 and April 2022. A total of 37,756 surgeries meeting the study criteria were included in the machine learning analysis. The study established a comprehensive perioperative database, integrating multi-source data from the Hospital Information System (HIS) and the Anesthesia Information Management System (AIMS), forming a multidimensional dataset to support in-depth analysis of high intraoperative blood pressure variability.

The "Hospital Database Management Procedures" consists of the following sections:

(1) Ethical Approval and Trial Registration;

(2) Database Management Procedures;

(3) Composition of the Original Database;

(4) Criteria for Inclusion of Model Features.

**(1) Ethical Approval and Trial Registration**

The design and execution of this study strictly adhered to the principles of medical research ethics and received approval from the Ethics Committee of Beijing Tsinghua Changgung Hospital (Approval No.: 22232-4-02). All patient data were sourced from the hospital's electronic medical records system, with data acquisition and usage conducted under the supervision and guidance of the hospital's Ethics Committee. Given that this is a retrospective study and all data were de-identified to protect patient privacy, the Ethics Committee waived the requirement for informed consent. Additionally, this study was registered on the ClinicalTrials platform, with the clinical trial identifier NCT05698433. During data processing, researchers strictly adhered to data security and privacy protection regulations, ensuring all patient information was anonymized and not traceable to specific individuals. Furthermore, all research activities complied with the internationally recognized Declaration of Helsinki and relevant domestic ethical regulations to ensure the compliance of the research activities.

**(2) Database Management Procedures**

To ensure the security and privacy of patient information, the hospital's information technology department implemented a series of data anonymization measures. Firstly, a bastion host was deployed within the regional network to enhance data security and ensure access control to the servers. This bastion host acts as a secure isolated node, managing all remote access requests to the servers, thereby adding an extra layer of security. Secondly, the complete dataset stored on the servers underwent a specially designed randomization process, which converts sensitive patient identifiers, such as medical record numbers and admission numbers, into irreversible unique sequence codes. This conversion process employs a high-strength random algorithm, ensuring that the sequence codes cannot be directly linked back to the original identifiers, thereby significantly reducing the risk of patient information leakage. Through these measures, the research team can maintain data integrity while effectively preventing the direct or indirect disclosure of patient personal information, ensuring the ethical compliance of the study.

**(3) Composition of the Original Database**

**Inpatient Visit Records (ANTHII_ADM_VISIT_MASTER file):** This file provides comprehensive baseline information about patients, including routine demographic data such as medical record number, gender, and age, as well as important details like admission date, discharge date, and the department the patient was admitted to. This information lays the foundation for subsequent risk stratification and cohort characteristic analysis.

**Diagnosis Records (ANTHII_DIAGNOSIS_RECORD file):** This file contains detailed records of all patient diagnoses, including standardized diagnosis codes and names. This information is crucial for identifying clinical conditions and complications associated with high intraoperative blood pressure variability (HIBPV).

**Anesthesia Events (ANTHII_MED_ANESTHESIA_EVENT file):** This file accurately records drug events during anesthesia, including drug names, dosages, administration routes, and administration times. It also includes the American Society of Anesthesiologists (ASA) classification of patients. The ASA classification system is widely used globally, where:

**ASA I:** A healthy patient with normal organ function.

**ASA II:** A patient with mild systemic disease without substantial functional limitations.

**ASA III:** A patient with severe systemic disease that limits activity but is not incapacitating.

**ASA IV:** A patient with severe systemic disease that is a constant threat to life.

**ASA V:** A moribund patient who is not expected to survive without the operation.

**ASA VI:** A declared brain-dead patient whose organs are being removed for donor purposes.

Including the ASA classification provides a simple, consistent, and easily understood method for assessing the risk of patients undergoing anesthesia and surgery.

**Primary Surgical Records (ANTHII_MED_OPERATION_MASTER file):** This file contains key information about surgeries, such as surgery time, anesthesia start and end times, surgery name, and type. This information is crucial for calculating surgery duration, assessing surgical complexity, and predicting surgery-related risks.

**Vital Signs Monitoring Data (ANTHII_MED_PAT_MONITOR_HISTORY file):** This file provides intensive vital signs monitoring data, including heart rate (HR), systolic blood pressure (SBP), diastolic blood pressure (DBP), mean arterial pressure (MAP), and respiratory rate.

**Laboratory and Examination Records (ANTHII_OBS_MASTER file, ANTHII_OBS_ITEMS file, ANTHII_OBS_MEASURE_REC file, ANTHII_OBS_SECTION_REPORT file):** These files contain detailed information about laboratory and examination processes, from request and execution to result reporting.

1. **Criteria for Inclusion of Model Features**

During the screening process for commonly used intraoperative medications, we focused on drugs that were administered to at least 10% of patients during surgery. This criterion helps reduce data heterogeneity arising from different types of surgeries. This screening standard allows us to concentrate on major categories of drugs, including vasoactive agents, anesthetics, sedatives, and analgesics, which are crucial for maintaining physiological stability, alleviating pain, and promoting sedation and anesthesia during surgery. Conversely, we intentionally excluded drugs with a usage frequency of less than 10%. This approach ensures that our data is more representative and practical, avoiding unnecessary variability introduced by marginally used medications. Additionally, considering that surgeons may use special or local medications based on specific intraoperative circumstances, such cases were excluded due to their highly individualized and infrequent nature. This decision further ensures the generalizability and standardization of our drug list, facilitating its application across various surgical settings while simplifying data processing and analysis.

Regarding preoperative diagnoses, we considered their impact on blood pressure variability, particularly in managing blood pressure-related conditions such as heart failure, renal failure, and hypertension. Diagnoses were made according to the International Classification of Diseases, Tenth Revision (ICD-10), established by the World Health Organization, which serves as a global standard for medical diagnoses, facilitating health management and clinical use worldwide. Heart failure is classified under ICD-10 code I50, with further subdivisions such as I50.0 (congestive heart failure, including right ventricular failure), I50.1 (left ventricular failure), and I50.9 (heart failure, unspecified). Renal failure diagnoses fall under codes N17 to N19, including N17 (acute renal failure), N18 (chronic renal failure), and N19 (renal failure, unspecified). Hypertension is coded from I10 to I15, encompassing I10 (essential hypertension) to I15 (secondary hypertension).

We included basic information such as age, height, weight, and gender to capture the potential impact of individual differences on intraoperative blood pressure variability. Age is a crucial factor in assessing cardiovascular risk during surgery. Weight and height are related to the patient's physiological state and circulatory system. Gender differences may also influence the patient's response to surgery and anesthesia. Preoperative laboratory test results, such as preoperative K^+^, Ca^2+^, tHB, Glu, serum creatinine (SCr), blood urea nitrogen (BUN), and albumin concentration (Alb), are commonly used indicators for evaluating preoperative organ function and metabolic status. Blood calcium and hemoglobin levels are directly associated with cardiovascular health, while creatinine and blood urea nitrogen reflect renal function, all of which are important factors in intraoperative blood pressure management. Preoperative diagnoses, including histories of hypertension, acute kidney injury, and stroke, provide crucial background information for intraoperative blood pressure variability. These medical histories not only reflect the patient's baseline health status but may also directly impact intraoperative blood pressure management. Additionally, the ASA classification, as a standardized tool for preoperative risk assessment, was included in feature selection. This classification system helps us understand the patient's overall health status and predict potential intraoperative risks, making it an important parameter for evaluating intraoperative blood pressure.

1. **Missing Data Cleaning Methods**

In addressing missing data, we first calculated the proportion of missing data for each variable and applied a 5% missing threshold as our feature selection criterion. Variables with missing data exceeding 5% were excluded from the analysis. For variables with ≤5% missing data, we employed mean imputation. In the application phase, variables exceeding 5% missing data were excluded from subsequent analyses, while for retained variables, missing values were imputed using the variable's mean. This approach balanced data integrity with analytical feasibility, effectively addressing missing data challenges while maximizing the retention of useful information.

Nine variables were affected by missing data: Lactate, SO_2_, Baseline Systolic Blood Pressure (SBP), Baseline Diastolic Blood Pressure (DBP), Weight, Height, Furosemide, Dexamethasone, and Hemoglobin (Hb).

The exact percentage of missing data for each variable ranged from 0.09% to 0.91%.

A detailed breakdown of the missing data for each variable, sorted from highest to lowest percentage, is as follows:

- Hemoglobin (Hb): 432 missing values (0.91%)
- Lactate：279 missing values (0.59%)
- SO_2_：43 missing values (0.09%)
- Baseline SBP: 321 missing values (0.68%)
- Baseline DBP: 321 missing values (0.68%)
- Height: 256 missing values (0.54%)
- Weight: 226 missing values (0.48%)
- Dexamethasone: 156 missing values (0.33%)
- Furosemide: 112 missing values (0.24%)
